# Cross-Sectional Study in a Large Cohort of Chinese Patients With *GJB1* Gene Mutations

**DOI:** 10.3389/fneur.2020.00690

**Published:** 2020-07-31

**Authors:** Xiaoxuan Liu, Xiaohui Duan, Yingshuang Zhang, Aping Sun, Dongsheng Fan

**Affiliations:** ^1^Department of Neurology, Peking University Third Hospital, Beijing, China; ^2^Department of Neurology, China–Japan Friendship Hospital, Beijing, China; ^3^Key Laboratory for Neuroscience, Ministry of Education/National Health Commission, Peking University, Beijing, China

**Keywords:** Charcot-Marie-Tooth disease, GJB1, CMTX1, genotype, phenotype

## Abstract

Charcot-Marie-Tooth (CMT) disease is a clinically and genetically heterogeneous group of inherited neuropathies. The *GJB1* gene is the pathogenic gene of CMTX1. In this study, we screened a cohort of 465 unrelated Chinese CMT patients from years 2007 to 2019 and 650 controls by direct Sanger sequencing in *GJB1* gene or targeted next-generation sequencing (NGS) or whole-exome sequencing (WES). A bidirectional Sanger sequencing would be performed on the 600 bases in the upstream promoter region and 30 bases in the 3′ untranslated region (UTR), if no mutation was found in the coding region of *GJB1* of the patient. According to the results, 24 missense mutations, 4 nonsense mutation, 1 entire deletion, 1 intronic mutation, and 4 frameshift mutations in *GJB1* were identified. Three of them were novel mutations (c.104 T>C, c.658-659 ins C, and c.811 del G). Moreover, central nervous system involvement was observed in five patients carrying mutations of R15W, V95M, R142W, R164W, and E186K. Our findings expand the mutational spectrum of the *GJB1* gene in CMT patients. We also explored the genotype–phenotype correlation according to the collected information in this study. NGS panels for detecting inherited neuropathy should cover the non-coding region of *GJB1*.

## Introduction

Charcot-Marie-Tooth disease (CMT) is a clinically and genetically heterogeneous inherited neuropathy (1), affecting ~1 in 1,214 people in the world (2). X-linked CMT (CMTX1) is the second most common form of CMT after CMT1A, accounting for 6.2% of all CMTs in the general population (2) and in 7–15% of all CMT patients in different patient cohorts (3–5). It is caused by Gap junction protein beta-1 [*GJB1*, also known as connexin 32 (*Cx32*)] gene mutations. The typical clinical feature of CMTX1 is characterized by a distal motor and sensory polyneuropathy with mixed demyelination and axonal degeneration. The onset age of male patients is earlier than that of females; the clinical features are also more severe in males.

To date, more than 450 mutations have been identified to be related to *GJB1* (hihg.med.miami.edu/code/http/cmt/public_html/index.html#/). The majority are missense mutations. Rare conditions like nonsense, gross deletion, frameshift, and mutations in non-coding regions have also been reported.

*GJB1* encodes a protein called Cx32. Cx32 belongs to a large connexin family that participates in the formation of intercellular gaps. It is distributed in the Schwann cells of the peripheral nerve (PNS) and oligodendrocytes in the central nervous system (CNS) (6). In myelinating Schwann cells, Cx32 localizes at the paranodes of non-compact myelin, providing a channel for the exchange of ions, and small molecules across the myelin sheath. The diverse functions of Cx32 include the transduction of electrical signals, growth control, and cell differentiation (7). The pathogenic mechanisms by which different *GJB1* mutations cause CMTX1 are not fully understood. The proposed mechanisms include loss of Cx32 function affecting the gap junctions (GJs) in the myelin sheath and causing CMTX1 peripheral manifestations (3, 8) or gain of function affecting the CNS (9).

In this study, Sanger sequencing in the *GJB1* gene, targeted next-generation sequencing (NGS), or whole-exome sequencing (WES), were performed in a large cohort of Chinese patients with CMT to investigate the frequency of *GJB1* mutations and expand the phenotype of CMTX1. The variants in the non-coding region of *GJB1* were defined for the first time in Chinese patients in this study. We also studied the genotype–phenotype correlation in these patients.

## Methods

### Patients

Four hundred and sixty-five pedigrees with CMT were enrolled in the Neurology Clinic Center of Peking University Third Hospital and China–Japan Friendship Hospital from 2007 to 2019. The patients were classified based on the clinical phenotype, mode of inheritance, and electrophysiological studies. The age of onset, clinical features, family history, CMT neuropathy score (CMTNS), and electrophysiological features of the patients were precisely collected. A CMTNS below 10 indicates patients are mildly affected, 10–20 indicates they are moderate affected, and above 20 means they are severely affected. Written informed consent was obtained from the patients and their parents for the publication of this report and any accompanying images. The study was approved by the Ethics Committee of Peking University Third Hospital (IRB 00006761).

### Mutation Analysis

**Figure 1:**
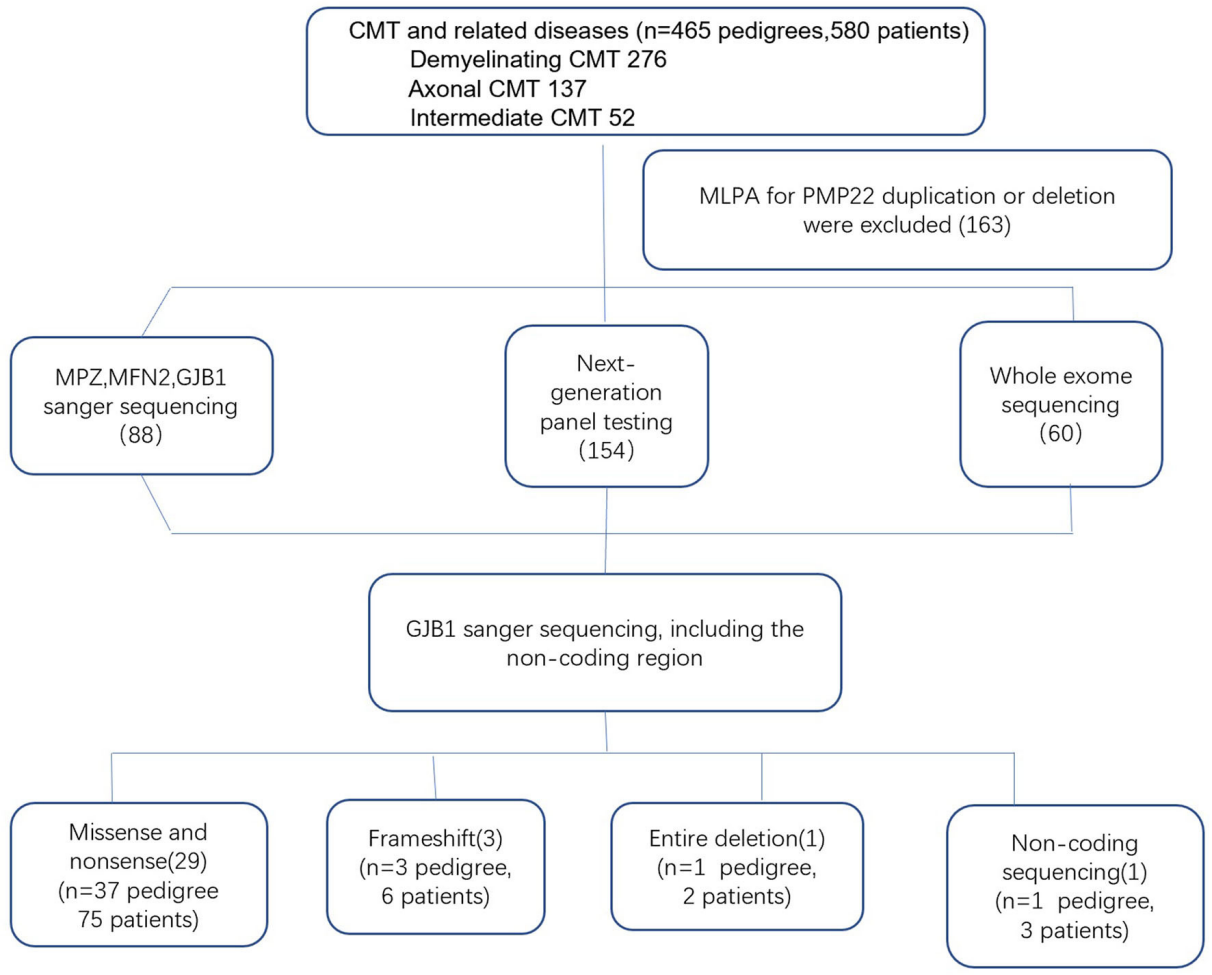
Genetic testing flowchart for patients with CMTX1.

Genomic DNA was extracted from the peripheral blood of the subjects using a DNA isolation kit (Bioteke, AU1802). Concentrations were determined on a Qubit fluorometer (Invitrogen, Q33216) using a Qubit dsDNA HS assay kit (Invitrogen, Q32851). Agarose gel (1%) electrophoresis was performed for quality control. By performing the multiplex ligation-dependent probe amplification (MLPA) technique in all patients with demyelinating and intermediate CMT, 163 index patients with *PMP22* duplications or deletions were initially excluded. Between 2007 and 2012, *GJB1* mutations were screened in 88 index patients by direct Sanger sequencing. After 2012, NGS gene panels covering 165 CMT and related disease genes were applied to the 154 index patients, and WES was performed in 60 index patients. All suspected variants were validated by Sanger sequencing (Figure 1). Analysis of *GJB1* mutation was based on the transcript version NM_000166.

#### Direct Sanger Sequencing

Sanger sequencing in exons of *GJB1* was performed. DNA extracted from the probands was amplified by polymerase chain reaction (PCR). The PCR products were then analyzed on an ABI 3730xl DNA analyzer (Applied Biosystems, Waltham, MA, USA), according to the manufacturer's protocol. If no mutation was detected in the coding regions, a bidirectional validation in the non-coding region of the 600 bases in the upstream promoter region and 30 bases in the 3′ untranslated region (UTR) would be performed.

#### Targeted NGS

Sample dilution, flow cell loading, and sequencing were performed according to Illumina specifications. DNA libraries were prepared with a KAPA library preparation kit (Kapa Biosystems, KR0453) following the manufacturer's instructions. The hybridization of pooled libraries to the capture probes followed by purification was carried out according to the Agilent SureSelectXT2 target enrichment system. Molecular analysis was established by a custom-designed targeted gene panel covering 165 genes. The bidirectional Sanger sequencing mentioned above was also designed and applied in company with the gene panel. Bidirectional validation was performed on the HiSeq 2500 platform.

#### WES

Agilent Human All Exon V6 kits were used for exome capture and sequenced on the HiSeq 2500 platform as paired-end 200-bp reads. Non-coding regions were not included in the WES. If no mutation was detected in the coding regions, a bidirectional Sanger sequencing in the non-coding region mentioned above was performed and validated in patients with suspected CMTX1.

### Pathogenicity Prediction

The assessment of the potential pathogenicity of the CMT mutations was performed using a standard method that included phenotype characterization and screening against Single Nucleotide Polymorphism Database (dbSNP) identifiers (http://www.ncbi.nlm.nih.gov/projects/SNP), the 1000 Genomes Project (http://www.1000genomes.org/), the Exome Aggregation Consortium (Exac), the ESP6500 database, and the Exome Variant Server (EVS) (http://evs.gs.washington.edu/EVS/) database; comparison with 650 Chinese controls; cosegregation with the phenotype in the available familial cases; and *in silico* pathogenicity prediction by SIFT (http://sift.jcvi.org/www/SIFT_enst_submit.html), PolyPhen (http://genetics.bwh.harvard.edu/pph2/index), and Mutation Taster (http://www.mutationtaster.org/). Variant classification was based on the ACMG standards (2013) (10).

## Results

### Demographics

Genotypic and phenotypic analyses in the cohort of 465 CMT families supportively diagnosed 276 CMT1, 137 CMT2, and 52 intermediate CMT families. Eighty-six patients with CMTX1 from 42 unrelated families who harbored 34 different *GJB1* mutations were evaluated. Based on the neurophysiology, the phenotypes of the 86 patients were classified to 71 intermediate CMT, 13 CMT1, and 2 CMT2. The mean age of onset was 18.3 years, ranging from 7 to 52 years. Sixty-one patients first exhibited symptoms during the first two decades of life. The proportion of male patients (67.4%, 58/86) was significantly higher than the proportion of female patients (32.6%, 28/86) (*P* < 0.05). The sex ratio was 2.1:1. Of the 42 index patients, 36 were males.

### Clinical and Electrophysiological Data

The initial symptoms included walking difficulties, muscle weakness in the distal lower limbs, and pes cavus or ankle joint contracture. Most patients (68/86, 79.1%) showed typical phenotypes, including symmetrical distal muscle weakness and atrophy with distal sensory loss and foot deformity. The mean CMTNS score was 15 (ranging from 0 to 32). The motor conduction velocity (MCV) was moderately reduced with a mean of 29.1–34.3 m/s in the median and ulnar nerves. Sensory nerve conduction velocity (SCV) was absent in 33.3% of the patients. The clinical manifestations and electrophysiological results of CMTX1 are shown in Table 1.

**Table 1:** Clinical features and electrophysiological results of all patients with GJB1 mutations.

	**Total (86)**	**Male (58)**	**Female (28)**
Age of onset (y), mean (SD, range)	18.3 (9.3, 7–52)	16.8 (9.8, 7–52)	20.8 (4.9, 12–24)
Family history, families (%)	36 (36/42, 87.8%)	/	/
Typical phenotype, *n* (%)	68 (79%)	50 (86.2%)	18 (64.3%)
Dexterity problems, *n* (%)	60 (70%)	48 (82.8%)	12 (42.9%)
Walking difficulty, *n* (%)	75 (87.2%)	55 (94.8%)	20 (71.4%)
Wheelchair dependent, *n* (%)	4 (4.7%)	3 (5.2%)	1 (3.6%)
Hearing problem, *n* (%)	8 (9.3%)	6 (10.3%)	2 (7.1%)
Pyramidal sign, *n* (%)	9 (10.5%)	7 (12%)	2 (7.1%)
CNS involvement	5 (5.8%)	5 (8.6%)	0
CMTNS (0–36)	15 (6.7, 0–32)	17.3 (7.5, 4–32)	13.5 (6.2, 0–24)
**MCV**			
Median CMAP (mv), mean (SD, range)	2.4 (2.0, 0–6)	2.1 (1.93, 0–6)	3.5 (2.4, 0.4–5.5)
Median MCV (m/s), (SD, range)	29.1 (11.1, 0–46.2)	28.3 (11.9, 0–46.2)	32.4 (6.7, 24.5–40.8)
Ulnar CMAP (mv), mean (SD, range)	3.8 (2.3, 0.7–10.7)	3.4 (2.5, 0.7–10.7)	4.6 (0.9, 0–6.7)
Ulnar MCV(m/s), (SD, range)	34.3 (7.7, 14.8–52.1)	32.8 (11.7, 26.4–44.7)	42.3 (8.5, 14.8–52.1)
**SCV**			
Median SNAP (uv), mean (SD, range)	3.3 (2.7, 0–7.3)	3.1 (2.6, 0–7.3)	3.6 (3.7, 0–6.5)
Median SCV(m/s), mean (SD, range)	25.2 (15.4, 0–38.2)	24.5 (15.1, 0–37.7)	28.1 (20.2, 0–38.2)
Ulnar SNAP (uv), mean (SD, range)	1.3 (1.5, 0–5.2)	1.1 (1.2, 0–1.79)	2.3 (2.7, 0–5.2)
Ulnar SCV(m/s), mean (SD, range)	22.9 (17.9, 0–43.5)	22.5 (18, 0–43.5)	24.9 (21.6, 0–38.2)
SCV absent, *n* (%)	28 (32.5%)	23 (39.6%)	5 (17.9%)

*CNS, central nervous system; CMTNS, Charcot-Marie-Tooth neuropathy score; MCV, motor conduction velocity; CMAP, compound muscle action potential; SCV, sensory conduction velocity; SNAP, sensory nerve action potential; CV, conduction velocity; /, not recorded*.

*Typical phenotype: slowly progressive distal muscle weakness, distal sensory loss, and depressed deep tendon reflex. Pyramidal sign: Babinski sign (+), Chaddock sign (+), or hyperreflexia were included. In nine patients with pyramidal sign, four had CNS involvement*.

When comparing the clinical characteristics between men and women, male patients often had a similar age of onset and disease progression rates, whereas female patients had diverse onset age and varied phenotypes ranging from asymptomatic to relatively severe. The age of onset was earlier in men than in women (16.8 vs. 20.8 years, respectively). Male patients were more likely than women to present dexterity problems (82.8 vs. 42.9%, respectively). The proportion of individuals with walking difficulties was higher in men than in women (94.8 vs. 71.4%, respectively). Nearly all patients were able to walk without an aid, and only a few patients required a wheelchair (5.2 vs. 3.6%; men vs. women). All patients with CNS involvement and white matter lesions were males. The CMTNS score was higher in male patients than in females (17.3 vs. 13.5, respectively). The median compound muscle action potential (CMAP) was lower in men (2.1 ± 1.9 vs. 3.5 ± 2.4 mv, respectively). The ulnar CMAP was also less in men (3.4 ± 2.3 vs. 4.6 ± 3.7 mv, respectively). The CMAP decline in males correlated with disease course (*r* = 0.456, *p* < 0.05). We also observed intermediate slowing of the MCV in median and ulnar nerves in both males and females (median 28.3 ± 11.9 vs. 32.4 ± 6.7 m/s, respectively; ulnar 32.8 ± 11.7 vs. 42.3 ± 8.5 m/s, respectively). A similar change tendency in SCV was also observed. The median nerve appeared to be more commonly affected than the ulnar nerve in both male and female patients.

Additional CNS involvement was also observed. Five patients had transient CNS involvement and white matter lesions. All patients with CNS involvement and white matter lesions were males. Four of the five patients with paroxysmal symptoms presented with a sudden onset of cerebral symptoms including aphasia, dysphagia, hemiplegia, or quadriplegia; the symptoms were induced by fever or diarrhea in three patients. One other case presented with vertigo and ataxia before the development of peripheral neuropathy. The symptoms often lasted for a few hours to a few days and resolved completely or partly without treatment. The cranial magnetic resonance images (MRI) of patient lesions involved the splenium and genu of the corpus callosum, bilateral posterior limbs of the internal capsule, centrum semiovale, and periventricular area. The cerebral demyelinating lesions were often symmetric, progressive, and enhanced with contrast without complete recovery. Two patients in our study had permanent CNS symptoms with transient white matter lesions, presenting hyperreflexia, and the Babinski sign. Eight patients (8/86, 9.3%) reported hearing problem and brainstem auditory evoked responses (BAEPs) abnormality. Six patients were males, and two were females.

Three novel *GJB1* variants were identified (c.104 T>C, c.658-659 ins C, and c.811 del G) in three families. The precise clinical features and electrophysiological data of patients were illustrated in Tables 2, 3. The proband of family 1,318(III-7) is a 45-year-old man who presented a severe early-onset motor and sensory neuropathy. Similar symptoms and age of onset were found in the patient's brothers and in his brothers' sons (III-1, III-8, III-11, III-12, and IV-7). The proband's daughter (IV-4) showed mild symptoms, and his brother's daughter (IV-5) showed only pes cavus. The heterozygous mutations c.104 T>C (p.V35A) was detected in all the affected patients in Family 1,318. A 33-year-old man in Family 1,312 suffered from symmetric weakness and atrophy in his hand from 15 years old. At the age of 26, he developed foot drop after a traumatic accident. These symptoms slowly progressed with unsteadiness worsening. His mother was reported to have minor weakness in the distal upper and lower limbs. A single-nucleotide insertion c.658-659 ins C (p.R220Pfs^*^23) was found in hemizygosity in the proband. The proband in Family 1,807 suffered from foot drop of his right foot since age 15 and developed foot drop of his left foot half a year later. He experienced muscle weakness and atrophy in the distal upper limb beginning at age 19. His mother had a milder phenotype and later onset age. His two deceased uncles (II-1 and II-2) had similar symptoms and onset age. His uncles' daughters (III-2 and III-4) had a mild phenotype. The proband's asymptomatic aunt had sons (III-5 and III-9) with the same *GJB1* mutation presenting moderate to severe phenotypes. A single-nucleotide deletion c.811 del G (p.A271Lfs^*^121) was found in hemizygosity in the proband.

**Table 2:** Clinical features of patients with novel mutations in *GJB1*.

**Patients**	**Sex**	**Onset age**	**Disease duration**	**Muscle strength** **(MRC score)**		**Muscle atrophy**		**Tendon reflexes**		**Sensory** **pinprick**		**Vibration sensation**		**CMTNS**	**Clinical features**
				**APB/ FDI/ ADM**	**Dorsiflexion/ plantarflexion**	**UL P/D**	**LL P/D**	**UL P/D**	**LL P/D**	**UL**	**LL**	**UL**	**LL**		
**1.1318**															
III-7	M	8	45	4/5/4	1/3	–/–	+/++	–/–	–/–	Below wrist	Below ankle	Below wrist	Below knee	21	Walking difficulty, pes cavus, tremor
IV-4	F	8	8	5–/5/5	3/4	–/–	–/+	+/+	+/–	No/no	No/no	No/no	Below ankle	10	Clumsy gait, hand atrophy
IV-5	F	14	6	5/5/5	5/5–	–/–	–/+	+/+	+/–	No/no	No/no	No/no	Below ankle	6	Pes cavus
IV-7	M	12	7	5–/5–/5–	3/3	–/–	+/++	+/+	+/–	No/no	No/no	Below wrist	Below ankle	14	Stepping gait, dexterity problem
**2.1312**															
III-2	M	10	23	5–/4/4	0/3	–/+	+/++	–/–	–/–	No/no	No/no	No/no	Below ankle	15	Weakness and atrophy in hand and feet
II-2	F	25	30	5–/5–/5–	4/3	–/+	+/++	+/+	+/–	Below wrist	Below ankle	Below wrist	Below ankle	12	Clumsy gait, hand atrophy
**3.1806**															
III-7	M	13	20	4/4/4	1/3	+/++	+/+++	+/+	+/+	Below wrist	Below knee	Below wrist	Below knee	17	Clumsy gait, hand atrophy, scoliosis
II-4	F	18	40	5/5/5	3/3	–/–	–/+	+/+	+/+	No/No	Below ankle	No/no	Below ankle	12	Clumsy gait

*APB, abductor pollicis brevis; FDI, first dorsal interosseous; ADM, abductor digiti minimi; UL, upper limb; LL, lower limb; P/D, proximal/distal; MRC, Medical Research Council*.

*Muscle atrophy: + mild atrophy; ++ moderate atrophy; +++ severe atrophy. Tendon reflex: +++ brisk; ++ normal; + reduced; – absent P/D. MRC score: 0 indicates no muscle movement, 1 indicates trace movement or fasciculation, 2 indicates movement against resistance without gravity, 3 indicates movement against gravity, 4 indicates movement against resistance but with reduced strength, and 5 indicates normal strength*.

**Table 3:** Neurophysiologic data of patients with novel mutations in GJB1.

**Patients**	**Sex**	**Compound muscle action potential**								**Sensory nerve action potential**						**EMG**
		**Median**		**Ulnar**		**Tibial**		**Peroneal**		**Median**		**Ulnar**		**Sural**		**ADM/TA**
		**Amp., mv**	**CV, m/s**	**Amp., mv**	**CV, m/s**	**Amp., mv**	**CV, m/s**	**Amp., mv**	**CV, m/s**	**SNAP, uv**	**CV, m/s**	**SNAP, uv**	**CV, m/s**	**SNAP, uv**	**CV, m/s**	
**1.1318**																
**III-7**	M	**0.81**	**38.5**	**1.69**	**33.2**	**Abs**	**Abs**	**Abs**	**Abs**	**Abs**	**Abs**	**Abs**	**Abs**	**Abs**	**Abs**	**+/+**
**IV-4**	F	5.2	**30.6**	**4.7**	**38.6**	**2.7**	**31.8**	**0.3**	**41.9**	**3**	**37.8**	**1.6**	**40**	**Abs**	**Abs**	**N/+**
**IV-5**	F	**4.1**	**30.8**	**4.2**	**38.6**	3.6	**31.5**	**1.3**	**38.7**	**3.2**	**37.5**	**4.2**	**34.8**	**2.9**	**36.1**	**+/+**
**IV-7**	M	**3.6**	**38.1**	**3.9**	**35.9**	**0.7**	**30.5**	**Abs**	**Abs**	**2.7**	**33.8**	**1.8**	**36.6**	**0.3**	**31**	**+/+**
**2.1312**																
**III-2**	M	**0.42**	**35.6**	**1.29**	**35.8**	**0.3**	**27.4**	**Abs**	**Abs**	**5.8**	**38.9**	**1.5**	**42.8**	**Abs**	**Abs**	**+/+**
**II-2**	F	5.5	**31.4**	**3.5**	**38.5**	**2.7**	**31.5**	**1.4**	**35.1**	**7.3**	**30.8**	**4.6**	**34.5**	**Abs**	**Abs**	**–/+**
**3.1806**																
**III-7**	M	**0.37**	**34.3**	**2.5**	**35.2**	**0.13**	**37.5**	**Abs**	**Abs**	**1.5**	**32.2**	**Abs**	**Abs**	**1.0**	**33.2**	**+/+**
**II-4**	F	9.6	51.5	5.5	59.6	**0.9**	**37.3**	**0.3**	**38.5**	**3**	**37.2**	**5.7**	**42.8**	**Abs**	**Abs**	**N/+**

*Amp, amplitude; CV, conduction velocity; SNAP, sensory nerve action potential; CMAP, compound muscle action potential; –, not recorded; Abs, absent; ADM, abductor digiti minimi; TA, tibialis anterior; +, neurogenic; N, normal value: CMAP (mv): median >5, ulnar >5, peroneal >3, tibial >3; MCV (m/s): median >50, ulnar >50, peroneal >40, tibial >40; SNAP (uv): median >7, ulnar >5, sural >6; SCV (m/s): median >45, ulnar >45, sural >40*.

*Bold values signify patients with abnormal value*.

### Mutation Analysis in *GJB1*

**Figure 2:**
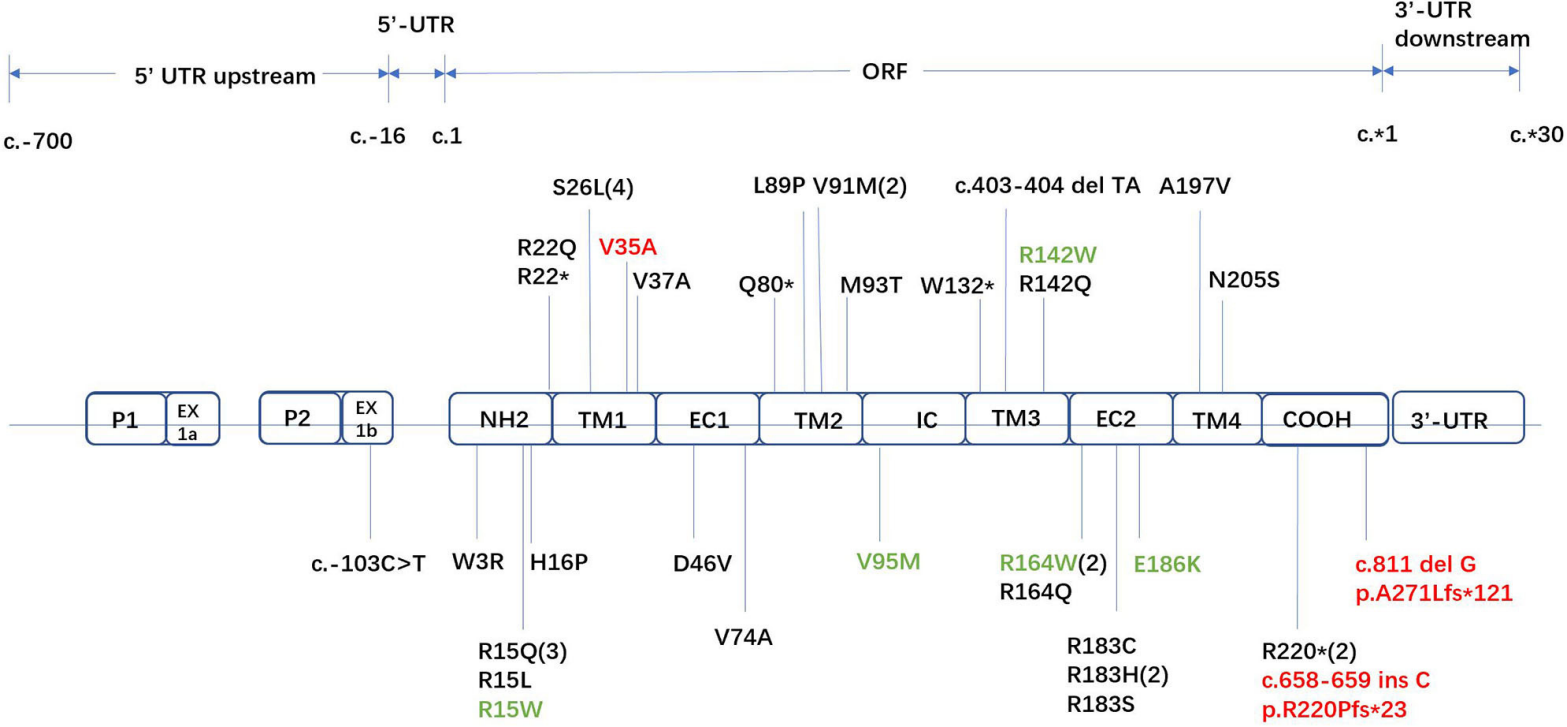
Locations of all *GJB1* mutations identified in our study. Novel mutations are marked in red. Mutations with transient white matter lesions are marked in green.

Forty-two unrelated families who harbored 34 different *GJB1* mutations were evaluated. Of the 34 mutations in *GJB1*, there are 24 missense mutations, 4 nonsense mutations (R22^*^, Q80^*^, W132^*^, and R220^*^), 1 gross deletion, 1 intronic mutation (c.103 C>T), and 4 frameshift mutations (three small deletions c.402 del C, c.403-404 del TA, and c.811 del G and one small insertion c.658-659 ins C) (Figure 2). Some unrelated patients shared identical mutations: R15Q(3), R22^*^(2), S26L(3), V91M(2), R164W(2), and R183H(2). Of these *GJB1* mutations, 1 mutation was located in the non-coding region, 10 mutations were located in the N-terminus, 5 mutations were located in the first transmembrane domain, 1 mutation was located in the first extracellular loop, 7 mutations were located in the second transmembrane domain, 1 mutation was located in the intracellular loop, 5 mutations were located in the third transmembrane domain, 8 mutations were located in the second extracellular loop, 1 mutation was located in the fourth transmembrane domain, 3 mutations were located in the C-terminus, and 1 was a gross deletion. The distribution of mutations was demonstrated in Figure 2. Regardless of the location and the type of the mutations, the clinical manifestations were similar in male patients. Even in patients with mutations in the 5′-UTR and entire deletions in *GJB1*, the clinical manifestations of the proband and other affected patients were not more severe than those of other patients in our cohort. CNS involvement and white matter lesions were identified in five patients (R15W, V95M, R142W, R164W, and E186K). These mutations are distributed in different regions of *GJB1*.

**Figure 3:**
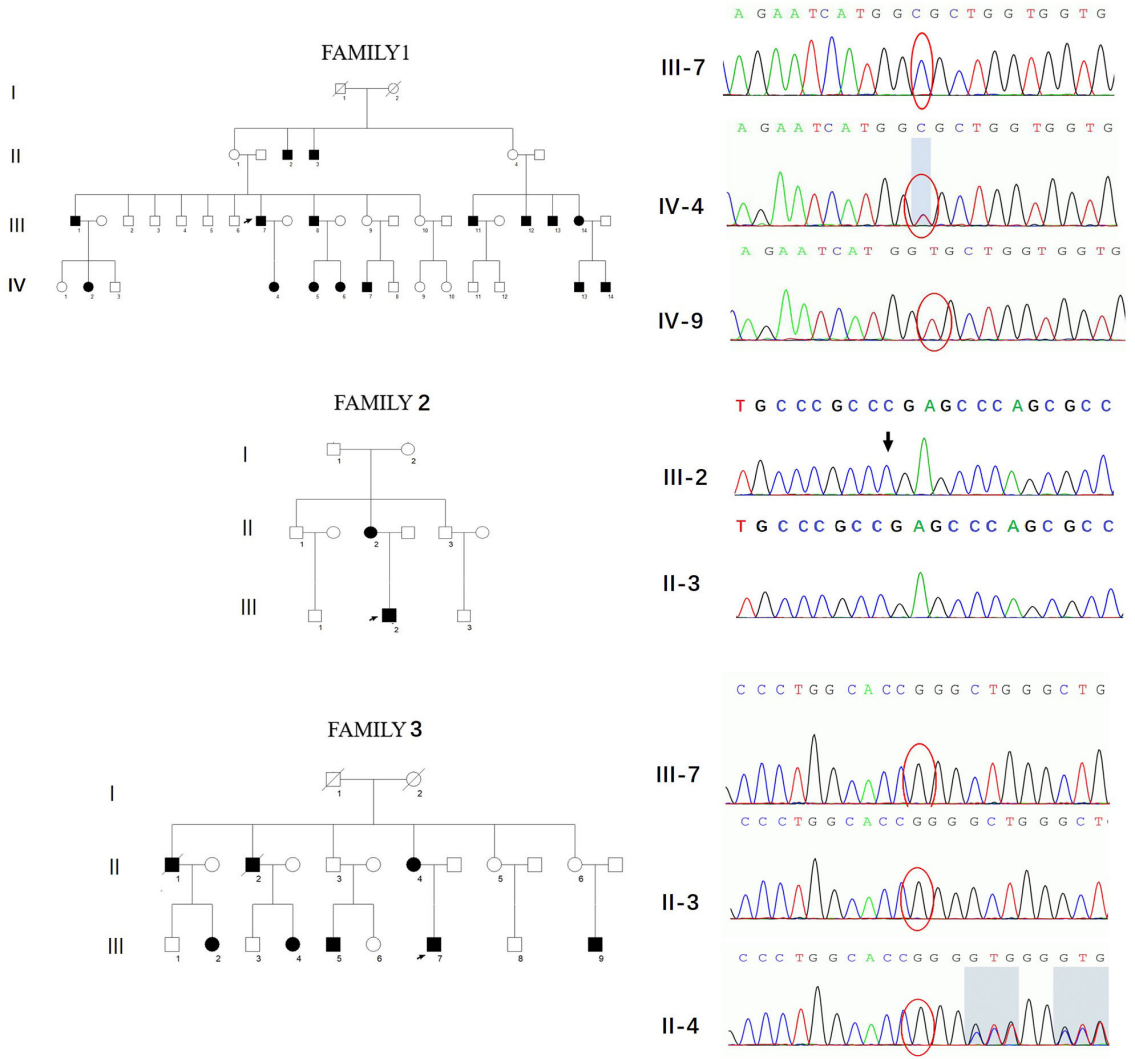
Pedigrees and genotypes of three families with novel *GJB1* mutations in our study.

Three variants in this study had not yet been reported (c.104 T>C, c.658-659 ins C, and c.811 del G). All these novel variants cosegregated with the phenotype, and they are absent in dbSNP129, Exac, and 1000 Genome project databases and also in 650 healthy controls in this study (PM2). The missense mutation, c.104 T>C, locates in the same locus as a pathogenic mutation (p.V35M) (PM5). Besides, this mutation cosegregated with disease in the family (PP1) and is PolyPhen-2 and deleterious by SIFT (PP3). Therefore, all three variants were classified as likely pathogenic according to the American College of Medical Genetics criteria (Figure 3, Table 4).

**Table 4:** Molecular analysis and predicted pathogenicity of novel variants in this study.

**Family**	**Chromosomal location**	**Nucleotide change**	**AA** **change**	**Effect**	**Database**			**Pathogenicity**			**GERP**	**Evidence**	**ACMG**
					**Exac**	**1000 G**	**650 controls**	**SIFT**	**PolyPhen-2**	**Mutation taster**			
1. 1318	Chr23: 70443661	c.104 T>C	p.V35A	TM1	0	0	0	D	Probably damaging	D	4.26	PM2, PM5, PP1, PP3	Likely pathogenic
2. 1312	Chr23: 70444215_70444216	c.658-659 ins C	p.R220Pfs*23	C-terminus	0	0	0	−	−	−		PVS1, PM2	Likely pathogenic
3. 1806	Chr23: 70444365	c.811 del G	p.A271Lfs*121	C-terminus	0	0	0	−	−	−		PVS1, PM2	Likely pathogenic

GERP is a software used to predict biological conservatism. A value more than two indicates more conservatism. **PVS1** is a very strong evidence of pathogenicity. It indicates a null variant (nonsense, frameshift, canonical ± 1 or 2 splice sites, initiation codon, and single or multiexon deletion) in a gene where loss of function is a known mechanism of disease. **PM2** is a moderate evidence of pathogenicity. It indicates absence from controls (or extremely low frequency if recessive) in the Exome Sequencing Project, 1000 Genomes Project, or Exome Aggregation Consortium. **PM5** is a moderate evidence of pathogenicity. It indicates novel missense change at an amino acid residue where a different missense change determined to be pathogenic has been seen before. **PP1** is a supporting evidence of pathogenicity. It indicates cosegregation with disease in multiple affected family members in a gene definitively known to cause the disease. **PP3** is a supporting evidence of pathogenicity. It indicates multiple lines of computational evidence supporting a deleterious effect on the gene or gene product (conservation, evolutionary, splicing impact, etc.).

## Discussion

Since the first report by Bergoffen et al. (11), over 450 *GJB1* mutations have been identified. Such mutations include missense, nonsense, frameshift, and deletion mutations (12). We found 34 different mutations in *GJB1*, including three that have not yet been reported. The frequency of *GJB1* mutations was 9% in this cohort of CMT patients from mainland China, which is similar to studies in American and European countries (5, 13–15), as well as studies in Asian countries, such as Japan and Korea, and other studies in China (3, 16–18).

The mutations identified in this study vary a lot in types and locations; however, the disease course of the male patients carrying these mutations is nearly identical. This indicates that these mutations have equivalent effects in protein function. In another large cohort in South China that identified 26 mutations in *GJB1* from 226 CMT families, the mutations were distributed in nearly all domains except for the first transmembrane domain and the distal C-terminus (3). Our results provide good supplement to enrich genotypic variability in China. To our knowledge, our research is the first study focusing on the 3′- and 5′-UTRs with such a large sample size in China. A recent study in the United Kingdom (19) revealed that the mutation in the non-coding region represented 11.4% of all cases of CMTX1. Though the mutation frequency in the non-coding area was not considerable in our study (1/34, 2.9%) and the c.103 T>C mutation has already been reported as one of the two most common mutations in the non-coding region (the other is −17 G>A), we still recommend performing the sequencing on the non-coding region of *GJB1* and adding the upstream and downstream coding sequences to the NGS panel. Three novel mutations have been identified in this study (c.104 T>C, c.658-659 ins C, and c.811 del G). The probands of the three families were all males and share similar and typical phenotypes, including juvenile onset, slowly progressive distal muscle weakness, sensory loss, and depressed deep tendon reflex. A neurophysiologic study showed intermediate slowing of NCV in median and ulnar nerves. Females tend to be affected much later and less severely than males. No CNS involvement was found in these patients with novel mutations. With a typical manifestation of peripheral neuropathy, together with a positive family history without male-to-male transmission, it is not difficult to consider the genetic diagnosis of *GJB1*. As previously reported, most patients shared common clinical characteristics, and male patients presented a more severe and rapid disease course with an earlier onset age compared with females. These phenomena were also observed in our study in many aspects, such as age of onset, dexterity problems, walking difficulty, CMTNS, and electrophysiological results (Table 1). We also observed that male patients often had similar onset age and disease progression rate, whereas women patients had varied phenotypes from asymptomatic to relatively severe. This was consistent with the previous study (15, 20) that found that approximately two thirds of female patients with *GJB1* mutations had a mild phenotype, one third had a moderately severe phenotype that progressed with time, and only a small proportion of patients remained in a subclinical state. Although it is not fully understood, the skewed X inactivation may partly explain the variable phenotype in females (4, 21) and has been documented in mice (22). In our cohort, five of the patients had transient CNS involvement and white matter lesions (R15W, V95M, R142W, R164W, and E186K). Each CNS manifestation was a variant combination of dysarthria, hemiparesis, cranial nerve deficits, motor aphasia, vertigo, and ataxia. Three patients had precipitating factors (R15W, R142W, and R164W), such as fever and diarrhea. The duration of CNS manifestation varies but usually resolves between a few hours and a few weeks. Two patients had permanent CNS symptoms (R15W and R142W), presenting hyperreflexia and the Babinski sign. It seems that CNS manifestations are not associated with the severity of peripheral neuropathy, and in one case, it is an initial manifestation of CMTX1. Eight patients (8/86, 9.3%) reported hearing problems and BAEP abnormality (R15W, R15L, M93T, V95M, R142W, R164W, E186K, and c.402 del C). Since Nicholson and Corbett first reported abnormal prolonged BAEPs in 1996 (23), five main CNS phenotypes have been described (4): (1) BAEP abnormality; (2) MRI abnormality without clinical manifestations; (3) transient CNS dysfunction; (4) mild to severe cognitive impairment; and (5) persistent CNS manifestations. In our study, no patient reported cognitive impairment, and the other four forms of CNS phenotypes can be observed, indicating that typical peripheral neuropathy with transient CNS involvement and hearing problems are good indicators of CMTX1.

*GJB1* encodes Cx32, a 283-amino-acid GJ protein that is highly expressed in Schwann cells and oligodendrocytes. Two hemichannels composed of six connexins form a GJ channel allowing transport of ions and small molecules. In PNS, Cx32 is localized to the non-compact myelin of incisures and paranodes (11), where it likely forms GJs between the layers of the Schwann cell myelin sheath (21). Clinical data suggest that loss of function of Cx32 may be sufficient to cause PNS disease, since the clinical manifestations of patients with nonsense and frameshift mutations in the N-terminus and those with a deletion of the entire coding sequence and the non-coding region that affects the *GJB1* promoter were similar to those observed in patients with other mutations. Many Cx32 mutants were proven to affect trafficking when expressed by transfection in mammalian cell lines (21). Other recent studies also identified dysfunction in specific permeability of important molecules with molecular masses of more than 1 kDa (7, 24). These might be the two main explanations of PNS involvement in CMTX1 patients.

In the CNS, Cx32 mutations affect both Cx32:Cx32 (oligodendrocyte–oligodendrocyte) coupling and Cx32:Cx30 (oligodendrocyte–astrocyte) coupling. The pathological mechanism of *GJB1* mutations affecting CNS still needs investigating; gain of function, loss of function, and their combination with environmental factors may play a role. There are some reasons to hypothesize that CNS manifestations may be due to gain of function. Although more than 30 mutations, including the five mutations identified in our study (R15W, V95M, R142W, R164W, and E186K), have been reported to be related to CNS impairment (4), these mutations only account for a small portion compared to the entire CMTX1 mutations. Secondly, patients with absence of the entire coding sequencing in our study and previous studies did not show any CNS involvement. Thirdly, transgenic mice expressing the R142W mutation on a background of wild-type Cx32 develop a demyelinating neuropathy not compensated by Cx32 overexpression (25). However, Abrams and colleagues compared the different mechanisms of “PNS + CNS” mutations (F51L, E102del, V139M, R142Q, R142W, R164W, T55I, R164Q, and C168Y) and “PNS-only” mutations (Y151C, V181M, R183C, and L239I), and they found that “PNS + CNS” mutations either could not form functional GJ plaques or produced little or no detectable junctional coupling in Neuro2A cells. Therefore, they postulated that the loss of function of CX32 could have a major effect on the pathogenesis of CNS disorders in CMTX1 (24). Further clinical manifestations are needed to validate the hypothesis of pathogenic mechanism of CNS in the future.

In conclusion, the frequency of *GJB1* mutations was 9% in this cohort of CMT patients from mainland China. The diagnosis of CMTX1 could be established in the intermediate slowing of NCV in patients with CMT and a positive family history with no male-to-male transmission, especially in those with transient CNS involvement or hearing problems. In our research, three novel likely pathogenic mutations were identified, which expand the variant diversity of CMTX1. Moreover, we also focused on the non-coding region of *GJB1* and successfully found one pedigree carrying an intronic variant. Therefore, the mutations in the intron were also suggested to be detected.

## Synopsis

Our cohort study is the first and largest to include the upper and lower non-coding regions of GJB1 in the NGS panel in a Chinese population. We also identified new GJB1 variants (c.104 T>C, c.658-659 ins C, and c.811 del G) and variants with transient CNS involvement (R15W, V95M, R142W, R164W, and E186K). Non-coding region mutations in Chinese individuals may be underestimated, and we recommend adding the upstream and downstream coding sequences to the NGS panel.

## Data Availability Statement

The datasets generated for this study can be found in the http://www.mono-mybg.com/jzjy-cmt.

## Ethics Statement

The studies involving human participants were reviewed and approved by The study was approved by the Ethics Committee of Peking University Third Hospital (IRB 00006761). Written informed consent to participate in this study was provided by the participants' legal guardian/next of kin. Written informed consent was obtained from the individual(s), and minor(s)' legal guardian/next of kin, for the publication of any potentially identifiable images or data included in this article.

## Author Contributions

DF and XL conceived and designed the study. XD provided valuable clinical materials. YZ and AS performed the genetic testing. XL wrote the paper. DF and XL reviewed and edited the manuscript. All authors read and approved the manuscript.

## Conflict of Interest

The authors declare that the research was conducted in the absence of any commercial or financial relationships that could be construed as a potential conflict of interest.
