# Supplementary material for: Cross-Sectional Study in a Large Cohort of Chinese Patients With GJB1 Gene Mutations
Source: Front Neurol. 2020 Jul 31;11:690. doi: 10.3389/fneur.2020.00690 (PMC7438869; doi:10.3389/fneur.2020.00690)
Supplement: Supplementary file 1 [file Table_1.DOCX]

**Supplement table 1 Molecular analysis of all index patients in this study**

| **No.** | **Nucleotide change** | **Protein change** | **Family No.** | **Patients No. (male, female)** | **Location** |
| --- | --- | --- | --- | --- | --- |
| **1.** | c.-103 C>T | - | 1509 | 3(2,1) | intron |
| **2.** | c.7 T>C | p.W3R | 1614 | 1(0,1) | NH2 |
| **3.** | c.43 C>T | p. R15W | 1541 | 2(1,1) | NH2 |
| **4.** | c.44 G>A | p. R15Q | 0801 | 1(1,0) | NH2 |
| **5.** | c.44 G>A | p. R15Q | 1101 | 1(1,0) | NH2 |
| **6.** | c.44 G>A | p. R15Q | 1722 | 1(1,0) | NH2 |
| **7.** | c.44 G>T | p. R15L | 1542 | 1(1,0) | NH2 |
| **8.** | c.47 A>C | p.H16P | 1824 | 7(3,4) | NH2 |
| **9.** | c.64 C>T | p. R22* | 1543 | 1(1,0) | NH2 |
| **10.** | c.64 C>T | p. R22* | 1904 | 4(2,2) | NH2 |
| **11.** | c.65 G>A | p. R22Q | 1430 | 1(0,1) | NH2 |
| **12.** | c.77 C>T | p. S26L | 1436 | 4(3,1) | TM1 |
| **13.** | c.77 C>T | p. S26L | 1530 | 2(1,1) | TM1 |
| **14.** | c.77 C>T | p. S26L | 1544 | 1(1,0) | TM1 |
| **15.** | c.104 T>C  (novel) | p. V35A | 1318 | 16 (11,5) | TM1 |
| **16.** | c.110 T>C | p. V37A | 1317 | 1(1,0) | TM1 |
| **17.** | c.221T>C | p. V74A | 1201 | 1(1,0) | EC1 |
| **18.** | c.238C>T | p.Q80* | 1503 | 2(0,2) | TM2 |
| **19.** | c.250G>C | p. V84L | 1940 | 1(1,0) | TM2 |
| **20.** | c.260C>G | p. P87R | 1859 | 2(1,1) | TM2 |
| **21.** | c.265C>G | p. L89V | 1744 | 1(1,0) | TM2 |
| **22.** | c.271G>A | p. V91M | 1519 | 2(1,1) | TM2 |
| **23.** | c.271G>A | p. V91M | 1738 | 1(1,0) | TM2 |
| **24.** | c.278T>C | p. M93T | 1102 | 1(1,0) | TM2 |
| **25.** | c.283G>A | p. V95M | 1701 | 4(2,2) | IC |
| **26.** | c.396G>A | p.W132* | 1775 | 1(1,0) | TM3 |
| **27.** | c.402delC | p.Y135Mfs*11 | 1922 | 1(1,0) | TM3 |
| **28.** | c.403-404 del TA | p. Y135Cfs*11 | 1545 | 1(1,0) | TM3 |
| **29.** | c.424C>T | p. R142W | 1404 | 1(1,0) | EC2 |
| **30.** | c.425G>A | p. R142Q | 1857 | 1(1,0) | EC2 |
| **31.** | c.490C>T | p. R164W | 1726 | 4(2,2) | EC2 |
| **32.** | c.490C>T | p. R164W | 1743 | 1(1,0) | EC2 |
| **33.** | c.491G>A | p. R164Q | 1505 | 3(3,0) | EC2 |
| **34.** | c.547C>T | p. R183C | 1546 | 1(1,0) | EC2 |
| **35.** | c.548G>A | p. R183H | 1547 | 1(0,1) | EC2 |
| **36.** | c.548G>A | p. R183H | 1605 | 1(1,0) | EC2 |
| **37.** | c.556G>A | p. E186K | 1737 | 1(1,0) | EC2 |
| **38.** | c. 614A>G | p. N205S | 1548 | 1(1,0) | TM4 |
| **39.** | c.658C>T | p. R220* | 1549 | 1(1,0) | COOH |
| **40.** | c.658-659 ins C  (novel) | p. R220Pfs*23 | 1312 | 2(1,1） | COOH |
| **41.** | c.811del G  (novel) | p. A271Lfs*121 | 1807 | 2(1,1) | COOH |
| **42.** | Gross deletion | - | 1720 | 1(1,0) | - |
